# Seventy-year long record of monthly water balance estimates for Earth’s largest lake system

**DOI:** 10.1038/s41597-020-00613-z

**Published:** 2020-08-21

**Authors:** Hong X. Do, Joeseph P. Smith, Lauren M. Fry, Andrew D. Gronewold

**Affiliations:** 1 School for Environment and Sustainability, University of Michigan, Ann Arbor, MI USA; 2 Faculty of Environment and Natural Resources, Nong Lam University, Ho Chi Minh City, Vietnam; 3 Cooperative Institute for Great Lakes Research (CIGLR), University of Michigan, Ann Arbor, MI USA; 4 Formerly Office of Great Lakes Hydraulics and Hydrology, United States Army Corps of Engineers, Detroit, MI USA; 5 NOAA Great Lakes Environmental Research Laboratory, Ann Arbor, MI USA

**Keywords:** Limnology, Hydrology

## Abstract

We develop new estimates of monthly water balance components from 1950 to 2019 for the Laurentian Great Lakes, the largest surface freshwater system on Earth. For each of the Great Lakes, lake storage changes and water balance components were estimated using the Large Lakes Statistical Water Balance Model (L2SWBM). Multiple independent data sources, contributed by a binational community of research scientists and practitioners, were assimilated into the L2SWBM to infer feasible values of water balance components through a Bayesian framework. A conventional water balance model was used to constrain the new estimates, ensuring that the water balance can be reconciled over multiple time periods. The new estimates are useful for investigating changes in water availability, or benchmarking new hydrological models and data products developed for the Laurentian Great Lakes Region. The source code and inputs of the L2SWBM model are also made available, and can be adapted to include new data sources for the Great Lakes, or to address water balance problems on other large lake systems.

## Abstract (summary)

**Table:** 

Measurement(s)	hydrological process • water balance
Technology Type(s)	computational modeling technique
Factor Type(s)	monthly water balance estimates
Sample Characteristic - Environment	lake
Sample Characteristic - Location	Great Lakes

Machine-accessible metadata file describing the reported data: 10.6084/m9.figshare.12764342

## Background & Summary

Among the most severe impacts of climate change is the intensification of the hydrologic cycle^1,2^. The Clausius-Clapyeron relation^3^, which defines specific humidity of the atmosphere as a function of temperature, suggests that the rising trend of global mean surface air temperature will lead to an increase in evaporation and precipitation^4^, and potentially exacerbate observed changes in river flows^5^, hydrological extremes^6,7^ and water availability^8,9^. These changes are particularly pronounced over Earth’s large lakes^10^ (which hold more than 90 percent of all global surface fresh water), where rapid increases in lake temperature^11^ have led to unprecedented water level dynamics on many of those lakes^12,13^. The intensified hydrologic cycle, coupled with the ever-increasing water demands of a rapidly growing population^14^, have strained global water resources, indicating a need for improved understanding of how the different components of the Earth’s system (e.g., climate, land surface, and human) have influenced the hydrologic cycle^15^. To meet this demand, hydrological models are often used^16^, in part because of their capacity to represent hydrologic variables across the global landmass. Model simulations have corroborated observed changes in components of the water cycle^17–19^, and related these changes to natural and anthropogenic factors^20,21^.

As hydrological models have become more advanced, simulations of water balance components (e.g. runoff, evaporation) have also been made available in the public domain^22–24^, providing opportunities to advance understanding of the hydrologic cycle at multiple spatiotemporal scales. However, uncertainties in global data products are often high, especially in regions with very large lakes^25^, as lake-atmospheric feedbacks can be challenging to simulate accurately^26,27^. To offset limitations of hydrologic model simulations, remote sensing data products are among the potential alternatives for large lakes research. Recent advances in remote sensing techniques^28^ have improved the accuracy of data products representing important variables of large lakes hydrology such as water levels^29^ and evaporation^30^. However, the development of remote sensing data sets usually does not take into account mass flux balance in the context of the overall hydrologic cycle. This limitation hinders the applicability for large lakes of remote sensing data sets, as they often cannot be used together with other independent data sources to explain the mechanisms driving changes in water storage^31^.

**Fig. 1:**
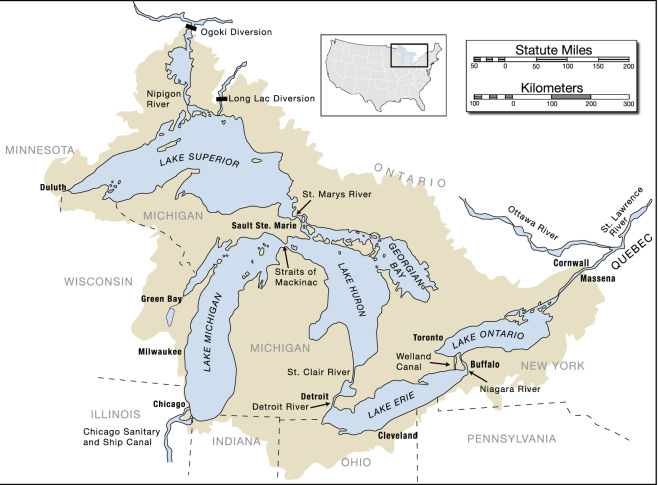
The main features of the Laurentian Great Lakes basin (shaded region) including lake surfaces (light blue), location of major cities, main inter-basin diversions, and connecting channels (Source: NOAA Great Lakes Environmental Research Laboratory and U.S. Army Corps of Engineers, Detroit District).

The Laurentian Great Lakes (hereafter referred to as the Great Lakes; Fig. 1), the largest system of freshwater lakes on Earth, represent many of the challenges facing global large lakes. Water levels of the Great Lakes have fluctuated in response to natural climate variability (e.g., variations in precipitation and evaporation) as well as direct anthropogenic factors such as regulation of outflows and inter-basin diversions^32–34^. The intensified dynamic of water levels in the last two decades^35^ has elevated societal concern of a potential new norm for the Great Lakes hydrologic cycle^12,36^ in the future as global temperatures continue to rise^37^, posing new challenges for regional water management. Although there are multiple data sources available to study the Great Lakes water balance^22,38–45^, none of them adequately quantify uncertainty^46,47^ or reconcile the water balance because they were developed independently.

To provide a framework for incorporating independent data sets and informing water management decisions for large lakes, a statistical framework (the Large Lakes Statistical Water Balance Model, hereafter referred to as the L2SWBM) has been recently developed^48,49^. This new model can assimilate independent data products to infer the value of water balance components through a Bayesian framework. A conventional water balance equation is used within the L2SWBM to constrain the estimates, ensuring that outputs can close the water balance over multiple time periods. The L2SWBM has been used to support Great Lakes hydrological research, particularly by attributing water level changes to climatic conditions^50^, assessing bias of different data products representing a common water balance component^47,51^, and benchmarking the performance of operational forecasts^31^.

This article presents a seventy-year record of Great Lakes water balance estimates using the L2SWBM. This dataset can be used to explore the mechanisms underlying long-term changes as well as the most recent surge of Great Lakes water levels, and provide new insight into how climate change has influenced, and might continue to influence large lakes. The inputs and source code of the L2SWBM are also made available, and can be customized to incorporate new measurements, estimates or simulations when they become available in the future.

## Methods

**Fig. 2:**
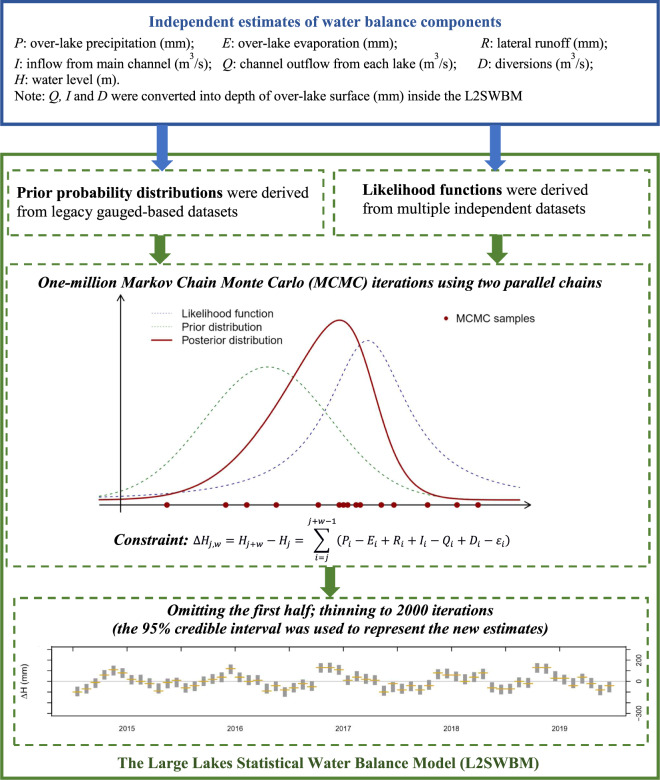
Schematic figure of the approach to generating new monthly estimate for the Great Lakes water balance.

Figure 2 shows a schematic of the Bayesian inference approach encoded in the L2SWBM. The following sections elaborate on the independent data sources used as inputs, and describe the components of the L2SWBM in greater detail.

### A compilation of multiple data sources for the Great Lakes water balance

Multiple hydro-climate datasets are available to represent the water balance of the Great Lakes^31^ ranging from gauge-based aggregated data^43,52^ to model simulations^47^ and remote sensing products^40^. However, they were mostly developed independently with limited consideration of fidelity to the water balance^31^. This inconsistency among available data sets is a long-standing challenge facing Great Lakes hydrologic research^41^, and has motivated the development of the L2SWBM^50^. To inform the Bayesian framework encoded within the L2SWBM, we selected eight independent data sources, including:

- Beginning of month (BOM) water levels (*H*) for each of the Great Lakes, provided by the binational Coordinating Committee on Great Lakes Basic Hydraulic and Hydrologic Data (referred to as “Coordinating Committee”, or CCGLBHHD, hereafter; for more information about this ad-hoc group, please see Gronewold, *et al*.^31^). Lake wide-average water levels were calculated as the arithmetic mean of daily water level measurements over a subset of *in situ* gauges located across the coastline of each lake. A greater detail of the underlying data sets is discussed in Gronewold, *et al*.^31^.
- Diversions (*D*) into, or out of, each lake were provided by the Coordinating Committee^31^. Diversions include the Long Lac and Ogoki Diversion into Lake Superior, the Chicago Diversion from Lake Michigan-Huron, and the Welland Canal, which diverts water from Lake Erie to Lake Ontario.
- Connecting channel flows (*I* or *Q*) are obtained from two independent data sources. The first dataset was estimated by the Coordinating Committee using a variety of methods such as stage-fall discharge equations or aggregation of discrete flow measurements^31^. The second dataset was measured using Acoustic Doppler Velocity Meters, which were installed across International Gauging Stations (IGS) maintained by the United States Geological Survey and Water Survey Canada^31^.
- Over-lake precipitation (*P*) is obtained from four data sources: (i) the NOAA-GLERL Great Lakes Monthly Hydrometeorological Database (GLM-HMD)^43^; (ii) output of the Great Lakes Advanced Hydrologic Prediction System (AHPS)^53^, which is operated by the United States Army Corps of Engineers (USACE); (iii) National Weather Service Multisensor Precipitation Estimates (NWS MPE)^54^; and (iv) Meteorological Service of Canada’s Canadian Precipitation Analysis (CaPA)^40,55^.
- Over-lake evaporation (*E*) is obtained from three data sources: (i) the NOAA-GLERL GLM-HMD^43^; (ii) output of the USACE AHPS^41^; (iii) the Environment and Climate Change Canada’s Water Cycle Prediction System (ECCC WCPS)^39^; and output of the NOAA-GLERL Finite-Volume Community Ocean Model (FVCOM)^56^.
- Tributary lateral runoff (*R*) is obtained from three data sources: (i) the NOAA-GLERL GLM-HMD^43^; (ii) output of the USACE AHPS^41^; and (iii) the ECCC WCPS^39^.

Table 1 provides a summary of these data sets and indicates which data set was used to estimate the prior distributions and likelihood functions. Besides the data sets included in Table 1, there are other regional^31^ and global^57,58^ data products that have been identified for potential applications of the L2SWBM on the water balance of the Great Lakes (and other large lakes) in the future.

**Table 1:** Summary of data sets, and an indication of which were used to calculate the prior probability distribution and likelihood functions, for each of the water balance components including over-lake precipitation (denoted as P), over-lake evaporation (denoted as E), lateral runoff (denoted as R), inflow through main channels from upstream lake (denoted as I), outflow through main channels (denoted as Q), diversion (denoted as D) and lake storage (denoted as H). Note that only data from 1950 to 2019 was used in this study.

Data sources	Variables	Temporal coverage	Used in		Data reference
			Prior distribution estimate	Likelihood function estimate	
NOAA GLERL GLM-HMD	*P, E, R*	1900–2016^(*)^	X	X	Hunter, *et al*.^43^
USACE AHPS	*P, E, R*	1900–2019^(*)^		X	Croley^53^
CCGLBHHD	*I, Q, D, H*	1900–2019	X	X	Gronewold, *et al*.^31^
IGS	*I, Q*	2008–2019		X	Gronewold, *et al*.^31^
GLERL FVCOM	*E*	2018–2019		X	Kelley, *et al*.^56^
ECCC CaPA	*P*	2006–2019		X	Fortin, *et al*.^40^ and Lespinas, *et al*.^55^
NWS MPE	*P*	2016–2019		X	Stevenson and Schumacher^54^
ECCC WCPS	*E, R*	2016–2019		X	Durnford, *et al*.^39^

^(*)^: over-lake evaporation is only available starting in 1949.

### The Large Lakes Statistical Water Balance Model (L2SWBM)

The L2SWBM uses a conventional water balance model to constrain component estimates, ensuring that the water balance can be closed over multiple timespans for the Great Lakes system. For Lake Superior, Lake Michigan-Huron, Lake Erie, and Lake Ontario, changes in storage over a specific time window were defined using Eq. (1).

1 $$\Delta {H}_{j,w}={H}_{j+w}-{H}_{j}=\mathop{\sum }\limits_{i=j}^{j+w-1}\left({P}_{i}-{E}_{i}+{R}_{i}+{I}_{i}-{Q}_{i}+{D}_{i}+{\varepsilon }_{i}\right)$$

where: Δ*H*: change in lake storage over *w* months, i.e. from month *j* to month *j* + *w* (mm);

*P*: over-lake precipitation (mm);

*E*: over-lake evaporation (mm);

*R*: lateral tributary lake inflow (mm);

*I*: inflow from upstream lake (m^3^/s);

*Q*: outflow to downstream lake (m^3^/s);

*D*: inter-basin diversions (to or from a specific lake) and consumptive uses (m^3^/s);

*ε*: process error term representing water level changes not explained by the other components (mm) such as ground-water fluxes or glacial isostatic rebound^59^.

We note that the L2SWBM code converts *I, Q*, and *D* from flow rate (m^3^/s) to lake-depth (mm) using lake surface area whenever required (e.g., the unit of millimetre is required to calculate water balance closure). The sign of *D* depends on whether water is diverted to (positive values) or from (negative values) a specific lake. In addition, this study used a rolling window of *w* = 12, which generally leads to better results regarding water balance closure^48,49^.

Over Lake St. Clair, which has a substantially smaller surface area relative to the other four lakes, the combined effect of inflow (from Lake Michigan-Huron via the St. Clair River) and outflow (to Lake Erie via the Detroit River) generally dominates the hydrologic cycle. Therefore, only net basin supply (*NBS* = *P* − *E* + *R*) was modelled, and the water balance equation for Lake St. Clair was modified as below.

2 $$\Delta {H}_{j,w}={H}_{j+w}-{H}_{j}=\mathop{\sum }\limits_{i=ji=j}^{j+w-1}\left(NB{S}_{i}+{Q}_{MH{U}_{i}}-{Q}_{i}+{\varepsilon }_{i}\right)$$

where *Q*_*MHU*_ is the outflow from Lake Michigan-Huron while the other variables are defined following those of Eq. 1.

Each water balance component was then inferred through a Bayesian approach, in which the “true” value of a variable (e.g., over-lake precipitation for Lake Superior) at a specific time-step (e.g., Jan 2019) was probabilistically estimated using a prior probability distribution and likelihood functions parameterized from multiple independent data sources. The following section will describe our approach to parameterizing the Great Lakes water balance using the L2SWBM. It is informative to note that the following sections share some similarity to the recent publication on the L2SWBM^48^. However, we also included more details on specific modifications (e.g., data used to derive the L2SWBM parameters) in our application to derive a seventy-year long record for the Great Lakes water balance.

#### Prior distributions of water balance components

We first modelled each water balance component with a probability distribution family, representing a “prior belief” of the possible range of values. The parameters of these distributions were empirically estimated from historical data spanning from 1950 to 2019 (presented in Table 1). Specifically, over-lake evaporation (*E*), connecting-channel inflow (*I*), connecting-channel outflow (*Q*), diversions (*D*) as well as net basin supply (NBS; for Lake St. Clair) corresponding to each calendar month *m* ($m\in [1,12]$) were modelled with a normal distribution:

3 $$\pi \left({E}_{m}\right)={\rm{N}}\left({\mu }_{E,m},{\tau }_{E,m}/2\right)$$

4 $$\pi \left({I}_{m}\right)={\rm{N}}\left({\mu }_{I,m},{\tau }_{I,m}\right)$$

5 $$\pi \left({Q}_{m}\right)={\rm{N}}\left({\mu }_{Q,{m}_{t}},{\tau }_{Q,m}\right)$$

6 $$\pi \left({D}_{m}\right)={\rm{N}}\left({\mu }_{D,{m}_{t}},{\tau }_{D,m}\right)$$

7 $$\pi \left(NB{S}_{m}\right)={\rm{N}}\left({\mu }_{NBS,m},{\tau }_{NBS,m}\right)$$

where the mean (*μ*) and precision (*τ*) parameters were calculated empirically from historical data. The use of the precision ($\tau =1/{\sigma }^{2}$) rather than the variance (${\sigma }^{2}$) in this study is the conventional practice for Bayesian inference^60^. We note that the precision of the prior probability distribution for *E* was divided by two (i.e., the variance was doubled) as showed in Eq. 3. This modification allowed a broader range of feasible values to account for a potential shift of evaporation in a warming climate^48^.

Lateral runoff (*R*) drained to each lake from the corresponding basin for each calendar month *m* ($m\in [1,12]$), which is always positive, was then modelled with a lognormal prior probability distribution:

8 $$\pi \left({R}_{t}\right)={\rm{LN}}\left({\mu }_{{\rm{ln}}(R),m},{\tau }_{{\rm{ln}}(R),m}\right)$$

where *t* is a specific time step, and prior mean (${\mu }_{{\rm{ln}}(R),m}$) and precision (${\tau }_{{\rm{ln}}(R),m}$) were calculated for each calendar month *m* using historical data records for that month. For example, at time step (*t*) January 2019, we have *m* equals 1 and the lateral runoff is modelled using mean and precision calculated from all observed January runoff values.

We modelled over-lake precipitation (*P*) using a gamma probability distribution, where the distribution parameters for each calendar month *m* were also calculated empirically from historical data.

9 $$\pi \left({P}_{m}\right)={\rm{Ga}}\left({\psi }_{m}^{1},{\psi }_{m}^{2}\right)$$

The shape (${\psi }^{1}$) and rate (${\psi }^{2}$) parameters of the gamma distribution were defined as below (following Thom^61^).

10 $${\psi }_{m}^{1}=\frac{1}{4{\phi }_{m}}\left(1+\sqrt{1+\frac{4{\phi }_{m}}{3}}\right)$$

11 $${\phi }_{m}=ln\left({\mu }_{P,m}\right)-{\mu }_{{\rm{ln}}(P),m}$$

12 $${\psi }_{m}^{2}={\psi }_{m}^{1}/{\mu }_{P,m}$$

where ${\mu }_{P,m}$ (Eq. 11), and ${\mu }_{{\rm{ln}}(P),m}$ (Eq. 12) are respectively the mean of historical precipitation, and the mean of the logarithm of precipitation for calendar month *m*.

The error term *ε* in Eq. 1 and Eq. 2 was also modelled using a vague normal prior probability distribution following Gelman^62^ across all calendar months:

13 $$\pi \left({\varepsilon }_{m}\right)={\rm{N}}\left(0,0.01\right)$$

#### Likelihood functions for analysis period

To derive the likelihood functions for the analysis period, data from multiple data sources spanning over the 1950–2019 period was used (note that the temporal coverage varies substantially across the data sets, as presented in Table 1).

For changes in lake storage over a period of *w* months, the likelihood function was defined as:

14 $${y}_{\Delta {H}_{j,w}}={y}_{{H}_{j+w}}-{y}_{{H}_{j}} \sim N\left(\Delta {H}_{j,w},{\tau }_{\Delta {H}_{j,w}}\right)$$

in which the observed change in storage over a rolling window of length *w* months (${y}_{\Delta {H}_{j,w}}$) is the difference between water level measurements (*y*_*H*_) at the beginning of month *j + w* and month *j*. We modelled this value with a normal distribution with mean $\Delta {H}_{j,w}$ and precision ${\tau }_{\Delta {H}_{j,w}}$.

The likelihood functions for water balance components on the right hand side of Eq. 1 (Eq. 2 for Lake St. Clair) follow a normal distribution:

15 $${y}_{t,\theta }^{n} \sim {\rm{N}}\left({\theta }_{t}^{n}+{\eta }_{\theta ,{m}_{t}}^{n},{\tau }_{t,\theta }^{n}\right)$$

where $\theta \in (P,E,R,I,Q,D,NBS)$, ${y}_{t,\theta }^{n}$ is data source $n\in [1,N]$ for component *θ* (which has *N* independent data sources) at time step *t* (e.g., *t* = Jan 2019; note that *m*_*t*_ = 1 in this case); ${\eta }_{\theta ,{m}_{t}}^{n}$ is the bias of data source number *n*^*th*^ in calendar month *m* ($m\in [1,12]$) and ${\tau }_{t,\theta }^{n}$ is the precision of data source number *n*^*th*^ at time step *t*.

Similar to other applications of the L2SWBM^50,51^, the precision of changes in lake storage (${\tau }_{\Delta {H}_{j,w}}$) and the precision of data sources of each water balance component over each time step (${\tau }_{t,\theta }^{n}$; $\theta \in P,E,R,I,Q,D,NBS$) were modelled with a gamma prior probability distribution with both shape and scale parameters equal 0.1:

16 $${\tau }_{\Delta {H}_{j,w}}={\rm{Ga}}\left(0.1,0.1\right)$$

17 $${\tau }_{t,\theta }^{n}={\rm{Ga}}\left(0.1,0.1\right)$$

Except for channel flows (of which the bias is relatively low for most lakes^46^), the bias of each contributing data set was modelled using a normal distribution with mean 0 and precision 0.01 (i.e., a standard deviation of 10):

18 $$\pi \left({\eta }_{\theta ,{m}_{t}}^{n}\right)={\rm{N}}\left(0,0.01\right)$$

#### Statistical inference of water balance components

To infer new estimates for the Great Lakes water balance over the 1950–2019 period, the L2SWBM was used to encode the prior distributions and likelihood functions estimated from available independent datasets into a JAGS (Just Another Gibbs Sampler) model inference routine^63^, which is an open-source successor to BUGS (Bayesian inference Using Gibbs Sampling)^64^. The ‘rjags’ package within the R statistical software environment^65^ was then used to simulate the JAGS model over 1,000,000 Markov Chain Monte Carlo (MCMC) iterations using two parallel MCMC chains. We omitted the first 500,000 iterations as a “burn-in” period. The remaining 500,000 iterations were then thinned at 250-iteration intervals to retain the final subset of 2,000 iterations. The 95% credible interval of the final subset was used to infer a feasible range for each water balance component.

It is informative to note that historical estimates of over-lake evaporation are not readily available from 1900 to 1949. As a result, this study only used data spanning from 1950 to 2019 to inform the statistical inference. To ensure that no observation was used to estimate both the prior distribution and the likelihood function (and thus would be favoured by the L2SWBM) at any specific time step, we used the following approach to derive the prior distributions:

- For the analysis period from 1950 to 1984: the prior distributions are generated from historical data covering the 1985–2019 period.
- For the analysis period from 1985 to 2019: the prior distributions are generated from historical data covering the 1950–1984 period.

## Data Records

The new estimates of the Great Lakes water balance, together with the L2SWBM source code and inputs synthesized for this project (monthly data available up to December 2019 depending on variables), are compressed as multiple zip-archives that are available for download^66^. The total file size of the dataset is approximately 4 MB, and contains:

(i) The L2SWBM source codes, stored in multiple R-script files, together with the R-script of the BUGS model and the model configuration file (accompanied with a text file explaining the variables of this configuration file). The configuration file can be adjusted to include more data sources or focus on a different analysis period. The abovementioned files were compressed into a zip archive named as “L2SWBM_Model.zip”.
(ii) Inputs of the L2SWBM. These independent data records were used to derive the prior distribution and likelihood functions for each of the variable. Data for each variable of a specific lake is stored in a separate csv file. All inputs files were compressed into a single zip archive named as “L2SWBM_input.zip”.
(iii) Outputs of the L2SWBM. The L2SWBM generated multiple outputs that are organized as four separate folders (each folder was compressed into one single zip archive). Table 2 provides a description of the data available as well as naming convention of these outputs.

**Table 2:** Description and naming convention of outputs generated by the L2SWBM.

Output	Type	Description	Naming Convention	Filename example
Prior distribution plots (output_plot_prior.zip)	Folder	Multiple PDF files that contain plots of the prior probability distributions of the water balance components	<VAR>PriorCompare_<PriorPeriod>.pdf	evapPriorCompare_19501984.pdf
Data-preview plots (output_plot_preview.zip)	Folder	Multiple PDF files that contain plots of inputs over the analysis period. Each pdf file shows independent data sources for a specific lake over one decade (from decade no. 0 to decade no. n-1, with n = no. of years/10).	<LAKE>TS_Preview_<DECADE No.>_<PROJECTNAME>.pdf	superiorTS_Preview_d0_GLWBData.pdf
Posterior inference plots (output_plot_posterior.zip)	Folder	Multiple PDF files that contain plots of outputs over the analysis period. Each pdf file shows all data for a specific lake over one decade (from decade no. 0 to decade no. n-1, with n = no. of years/10).	<LAKE>TS_ALL_<DECADE No.>_<PROJECTNAME>.pdf	miHuronTS_ALL_d5_GLWBData.pdf
Posterior inference time-series (output_ts_posterior.zip)	Folder	Multiple CSV files that contain monthly inference (2.5, 50 and 97.5 percentile of the MCMC iterations) of each water balance component across each lake over the analysis period.	<LAKE><VAR>_<PROJECTNAME>.csv	erieRunoff_GLWBData.csv

“Naming Convention” field represents the naming convention of individual files within a specific folder (compressed into a zip archive).

## Technical Validation

**Fig. 3:**
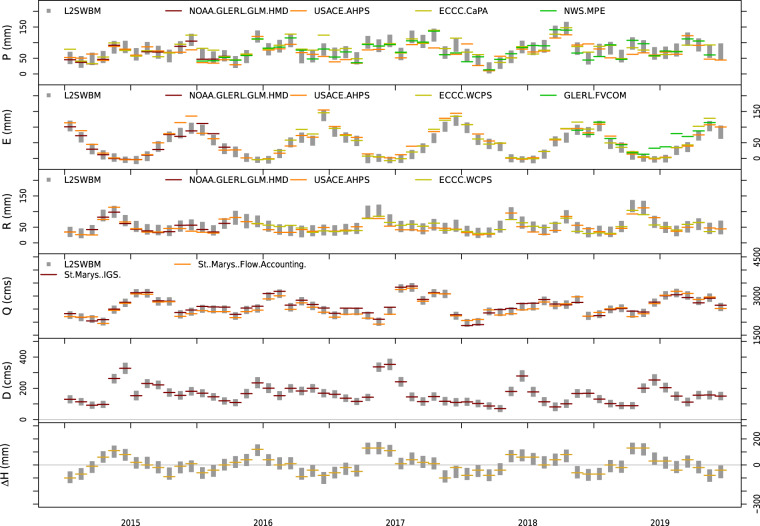
Comparison between the newly-derived water balance components generated by the L2SWBM (vertical grey bars) and corresponding observations from independent data sets (horizontal dashes) for Lake Superior from 2015 to 2019. From top to bottom: over-lake precipitation (denoted as P), over-lake evaporation (denoted as E), lateral runoff (denoted as R), outflow (denoted as Q), diversions (denoted as D) and changes in lake storage (denoted as Δ*H*). All of the included data sets are made available in Do, *et al*.^66^. Figures for each of the decadal periods (e.g., 1950–1959 or 1960–1969) across all lakes are also available in Do, *et al*.^66^.

Figure 3 provides a visual assessment of a representative time series of inferred values (95% credible interval of L2SWBM simulations) of storage changes and water balance components for Lake Superior from 2015 to 2019. We note that the published dataset^66^ also contains the graphs for each of the decadal periods (e.g., 1950–1959) across all lakes. The results in Fig. 3 (and other figures in Do, *et al*.^66^) indicate the presence of important differences among the historical data sets. For instance, there are substantial differences between over-lake precipitation (the top panel) aggregated from ECCC CaPA gridded product and that available in a legacy dataset (USACE AHPS).

The Bayesian inferred values (the vertical grey bars in Fig. 3) show generally consistent seasonal and inter-annual patterns, but also contain important differences relative to the other datasets. We also note that estimates over downstream lakes (i.e., Lake Erie and Lake Ontario) show generally higher uncertainty relative to the upstream lakes (i.e., Lake Superior and Lake Michigan-Huron), potentially due to the accumulation of uncertainty of the model simulations.

It is informative to note that the bias in channel flows (i.e., inflow, outflow and diversion) was not modelled by the L2SWBM, owing to relatively reliable records of river discharge comparing to estimates of other variables such as over-lake precipitation^46^. In addition, only one data source is available to represent changes in lake storage. As a result, estimates of channel flows and lake storage changes appear to be well constrained across all time steps relative to the other variables. Moving forward, we plan to assimilate new global data products of water level (e.g. water level derived from Gravity Recovery and Climate Experiment data^57^), and river flow simulated by hydrological models (e.g. WRF-Hydro model^67^) to provide a more holistic view of the uncertainty associated with L2SWBM estimates of these variables.

Tables 3–6 provide an overview of the new estimates of over-lake precipitation, over-lake evaporation, and lateral runoff across Lake Superior (Table 3), Lake Michigan-Huron (Table 4), Lake Erie (Table 5), and Lake Ontario (Table 6). Here we calculated the mean and the standard deviation of the median (denoted as MED) and the 95% credible interval (denoted as CI) estimated by the L2SWBM for each calendar month. Over-lake precipitation tends to have the highest inter-annual variations (indicated by a high standard deviation of MED) in Lake Superior and Lake Michigan-Huron, while lateral runoff has the highest inter-annual variation in Lake Erie and Lake Ontario, of which the ratio of land area to lake area is relatively high^32^.

**Table 3:** The mean and standard deviation (values inside the brackets) of the median (denoted as MED) and the 95% credible interval (denoted as CI) of the L2SWBM inference for over-lake precipitation (denoted as P), over-lake evaporation (denoted as E), and lateral runoff (denoted as R) over Lake Superior. The mean and standard deviation were calculated for each calendar month.

	Jan	Feb	Mar	Apr	May	Jun	Jul	Aug	Sep	Oct	Nov	Dec
*P MED*	52 (17)	36 (15)	43 (20)	53 (25)	72 (24)	79 (24)	75 (26)	76 (28)	86 (30)	76 (30)	64 (23)	56 (17)
*P CI*	22 (3)	21 (2)	22 (3)	23 (2)	24 (3)	24 (1)	24 (2)	25 (2)	25 (2)	25 (2)	24 (2)	23 (3)
*E MED*	100 (15)	59 (16)	41 (14)	16 (6)	2 (2)	-3 (1)	-1 (3)	13 (10)	48 (16)	70 (14)	96 (16)	114 (19)
*E CI*	19 (1)	19 (1)	18 (0)	15 (1)	11 (0)	8 (3)	13 (2)	17 (2)	19 (1)	18 (1)	19 (2)	21 (2)
*R MED*	33 (5)	30 (5)	40 (9)	88 (25)	93 (33)	58 (16)	44 (13)	35 (10)	36 (13)	47 (17)	45 (12)	38 (9)
*R CI*	16 (2)	15 (2)	17 (1)	22 (1)	24 (1)	20 (1)	18 (1)	17 (1)	18 (2)	19 (2)	19 (1)	17 (2)

**Table 4:** The mean and standard deviation (values inside the brackets) of the median (denoted as MED) and the 95% credible interval (denoted as CI) of the L2SWBM inference for over-lake precipitation (denoted as P), over-lake evaporation (denoted as E), and lateral runoff (denoted as R) over Lake Michigan-Huron.

	Jan	Feb	Mar	Apr	May	Jun	Jul	Aug	Sep	Oct	Nov	Dec
*P MED*	55 (18)	43 (17)	52 (23)	70 (22)	73 (26)	77 (27)	73 (19)	80 (21)	85 (32)	77 (31)	70 (23)	62 (20)
*P CI*	19 (2)	18 (2)	19 (2)	20 (1)	21 (2)	21 (1)	20 (2)	19 (3)	22 (2)	21 (2)	20 (2)	20 (2)
*E MED*	75 (14)	40 (11)	28 (10)	9 (5)	0 (3)	-1 (3)	8 (9)	33 (13)	61 (16)	77 (16)	94 (17)	105 (18)
*E CI*	15 (2)	13 (2)	13 (4)	10 (2)	8 (1)	8 (1)	12 (2)	14 (3)	15 (3)	15 (3)	16 (2)	16 (3)
*R MED*	58 (16)	53 (15)	84 (22)	115 (32)	87 (28)	54 (18)	38 (11)	31 (5)	34 (12)	47 (19)	59 (20)	63 (19)
*R CI*	20 (3)	20 (3)	22 (3)	24 (2)	23 (3)	20 (3)	18 (3)	15 (2)	18 (3)	20 (3)	21 (3)	21 (4)

**Table 5:** The mean and standard deviation (values inside the brackets) of the median (denoted as MED) and the 95% credible interval (denoted as CI) of the L2SWBM inference for over-lake precipitation (denoted as P), over-lake evaporation (denoted as E), and lateral runoff (denoted as R) over Lake Erie.

	Jan	Feb	Mar	Apr	May	Jun	Jul	Aug	Sep	Oct	Nov	Dec
*P MED*	63 (26)	53 (24)	66 (25)	80 (26)	79 (30)	84 (31)	80 (25)	81 (30)	84 (32)	78 (33)	79 (30)	73 (24)
*P CI*	23 (6)	23 (5)	23 (5)	24 (5)	25 (4)	25 (4)	24 (5)	25 (5)	26 (5)	25 (6)	24 (5)	24 (6)
*E MED*	42 (11)	22 (9)	17 (6)	7 (6)	14 (11)	32 (11)	71 (15)	111 (15)	155 (25)	179 (25)	139 (23)	92 (16)
*E CI*	15 (3)	14 (4)	12 (3)	12 (3)	14 (4)	15 (2)	16 (3)	16 (3)	19 (2)	19 (4)	19 (2)	17 (4)
*R MED*	95 (63)	97 (52)	142 (55)	123 (40)	75 (39)	50 (28)	34 (20)	25 (12)	28 (19)	37 (27)	63 (38)	90 (50)
*R CI*	42 (6)	42 (6)	45 (5)	43 (5)	41 (6)	38 (7)	34 (8)	30 (7)	32 (8)	35 (8)	40 (7)	43 (6)

**Table 6:** The mean and standard deviation (values inside the brackets) of the median (denoted as MED) and the 95% credible interval (denoted as CI) of the L2SWBM inference for over-lake precipitation (denoted as P), over-lake evaporation (denoted as E), and lateral runoff (denoted as R) over Lake Ontario.

	Jan	Feb	Mar	Apr	May	Jun	Jul	Aug	Sep	Oct	Nov	Dec
*P MED*	65 (21)	56 (22)	60 (22)	73 (24)	73 (29)	75 (31)	68 (22)	75 (21)	80 (28)	79 (33)	78 (24)	75 (22)
*P CI*	24 (3)	23 (3)	23 (3)	24 (2)	24 (3)	25 (3)	24 (2)	24 (3)	25 (4)	25 (4)	24 (3)	24 (3)
*E MED*	99 (17)	55 (14)	39 (11)	13 (7)	3 (5)	9 (9)	34 (14)	62 (12)	78 (14)	82 (15)	87 (17)	111 (21)
*E CI*	14 (1)	13 (1)	11 (2)	10 (2)	9 (1)	11 (1)	14 (2)	13 (2)	13 (2)	13 (3)	14 (1)	16 (2)
*R MED*	158 (60)	147 (60)	252 (74)	299 (88)	172 (75)	91 (38)	61 (29)	48 (16)	58 (30)	97 (47)	143 (60)	174 (58)
*R CI*	39 (20)	39 (18)	42 (18)	44 (17)	42 (18)	35 (19)	31 (19)	26 (15)	30 (18)	37 (20)	40 (20)	41 (20)

The CI of the new estimates is generally consistent across all time steps, indicated by a relatively small value of both the mean and the standard deviation. Our calculated uncertainties in each water balance component are susceptible to both the a priori range of values for that component, and to the range of variability in assimilated estimates. Consequently, some estimates, such as over-lake evaporation, can have low uncertainty values because evaporation has a very strong seasonal cycle, with very low values in the summer months. In future research, we intend to experiment with different expressions of the a priori water balance uncertainty to determine whether they impact the uncertainties of the L2SWBM estimates.

**Fig. 4:**
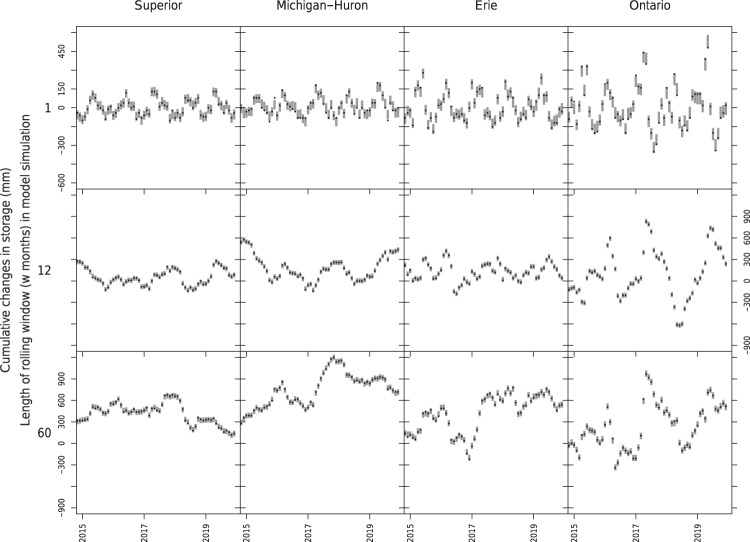
Water balance closure assessment using our new L2SWBM water balance estimates across the Great Lakes from 2015 to 2019. Vertical grey bars represent simulated cumulative changes (95% posterior predictive intervals) while black points represent observed cumulative changes in storage over one month (top panels), 12 month (middle panels), and 60 month periods (lower panels). Note that the range of the y axis varies across different rolling windows.

To assess long-term water balance closure, we also compared the cumulative changes in lake storage simulated by the L2SWBM with those obtained from observed data. Figure 4 shows the results of this comparison over the 2015–2019 period, indicating the capacity of L2SWBM estimates to close the water balance over consecutive periods of 1-, 12-, or 60-months.

The ability of the new estimates to reconcile the water balance provides a potential pathway towards improved understanding of hydrologic response to long-term climate variability. In addition, the uncertainties of water balance components inferred through the new estimate could be used to identify the time windows that need additional information such as new simulations using state-of-the-art hydrological models.

## Data Availability

The statistical model (L2SWBM) used to produce the new estimate for Great Lakes water balance was programmed in R (version 3.6.1). The scripts are open source and available for download as part of the published dataset^66^.
